# The Missing Retention Agenda: A Commentary on the EU‐Funded Nursing Action Initiative

**DOI:** 10.1111/jnu.70066

**Published:** 2026-03-23

**Authors:** Rosario Caruso, Alessandro Stievano

**Affiliations:** ^1^ Health Professions Research and Evidence Transfer Unit IRCCS MultiMedica Sesto San Giovanni Italy; ^2^ Department of Biomedical Sciences for Health University of Milan Milan Italy; ^3^ European Specialist Nurses Organization Arnhem the Netherlands; ^4^ Department of Clinical and Experimental Medicine University of Messina Messina Italy

**Keywords:** EU health policy, nursing workforce sustainability, retention governance, safe staffing, workforce planning

## Abstract

**Purpose:**

To critically examine the policy logic of the EU‐funded WHO Nursing Action Initiative and assess its capacity to address the structural drivers of Europe's nursing workforce instability, with a specific focus on retention governance as the missing determinant of sustainability.

**Organizing Construct or Argument:**

Although Europe reports high aggregate numbers of nurses, persistent workforce shortages are driven not by insufficient supply but by systemic governance weaknesses that undermine retention. The Nursing Action Initiative provides the first coordinated, multi‐country framework aligned with the WHO's 2023–2030 strategic priorities, yet several structural gaps, including the absence of binding retention metrics, enforceable safe staffing standards, harmonized advanced practice pathways, interoperable workforce intelligence, and mandatory accountability, limit its transformative potential. A shift from production‐centric policies to a retention‐driven governance architecture is therefore essential.

**Conclusions:**

The Nursing Action Initiative represents an important step toward strengthening European nursing workforce policy, but its success will depend on Member States' willingness to implement structural reforms that ensure safe staffing, protect nurses' well‐being, expand autonomous practice roles, and stabilize workforce distribution. Without a robust architecture of retention governance, neither the sustainability of Europe's nursing workforce nor the resilience of its health systems can be assured.

**Relevance:**

This commentary advances the policy debate by framing retention as the central determinant of workforce sustainability. It calls for urgent political commitment to move the Nursing Action Initiative beyond aspirational coordination and toward enforceable, system‐level reform capable of delivering lasting improvements in workforce stability and quality of care across the European Union.

## 1 Introduction

Given the severity and persistence of the nursing workforce shortage across Europe, this commentary is presented as the first in a series of invited commentaries examining complementary policy approaches to support, retain, and sustain the nursing workforce. Europe stands at a paradoxical juncture in its nursing workforce landscape. According to the World Health Organization (WHO) Europe, the Region has the highest density of health and care workers globally. Yet, it is simultaneously experiencing some of the most severe and persistent workforce gaps. This contradiction reflects a structural fragility rather than a numerical insufficiency: despite high aggregate numbers, the system is failing to retain, protect, and strategically deploy its nursing workforce (WHO 2025).

The WHO's Framework for Action on the Health and Care Workforce in the WHO European Region 2023–2030 underscores the multiplicity of pressures eroding workforce sustainability (WHO 2023). A rapidly aging nursing workforce, combined with a suboptimal and often rigid skill mix, is narrowing the pool of nurses able to meet evolving population health needs. Retention has emerged as a central vulnerability, with many countries reporting an acceleration of exits from the profession, fueled by chronic understaffing, moral distress, inadequate recognition, and limited career progression. Compounding these dynamics is the intensification of cross‐border mobility within the EU. Wage disparities, better working conditions, and clearer career pathways in high‐resource countries are driving substantial outmigration from lower‐resource settings, deepening regional inequities and destabilizing already fragile health systems (Greenley et al. 2024). The situation is further aggravated by the cascading effects of burnout, sustained stress after the pandemic, and increasing concerns about workplace safety, including exposure to violence (Vrbnjak et al. 2025). Each of these factors exerts a cumulative, mutually reinforcing impact on nurses' intention to leave, a pattern well documented in European workforce studies.

Beyond its immediate impact, the COVID‐19 pandemic functioned as a systemic stress test for European nursing workforces, exposing long‐standing deficiencies in staffing safety, psychosocial protection, and crisis preparedness (Stievano et al. 2024). In many countries, the absence of resilient retention safeguards during the pandemic accelerated moral injury, burnout, and premature exits from the profession. These lessons highlight that future workforce sustainability depends not only on emergency response capacity but also on embedding retention‐oriented governance mechanisms capable of protecting nurses during large‐scale shocks and comparable health system emergencies.

These trends reveal a critical insight: workforce density figures mask profound underlying vulnerabilities. The presence of large numbers of registered nurses does not guarantee system resilience if retention rates collapse, if distribution is unequal, or if working conditions erode the profession's capacity to function effectively. Europe's shortage, therefore, is not fundamentally a supply deficit but a manifestation of systemic governance weaknesses, including poor planning, inadequate investment, weak protection, and insufficient attention to the lived realities of nursing work. As the EU‐funded WHO Nursing Action Initiative accelerates, this turning point requires a decisive shift in policy logic: from a narrow focus on producing more nurses to a comprehensive strategy that prioritizes keeping nurses in the profession, ensuring their safety, enabling their autonomy, and valuing their contributions (Greenley et al. 2024; WHO 2025).

Despite growing awareness of these challenges, European policy responses continue to overemphasize workforce production while underestimating the structural drivers of premature exit from the profession (Stievano et al. 2024). Critical issues such as unsafe staffing, skill underutilization, gender‐based inequities, and deteriorating psychosocial conditions remain insufficiently addressed in current governance frameworks. The launch of the EU‐funded WHO Nursing Action Initiative is therefore a timely and important step. Still, its success needs to be critically examined against the fundamental determinants of workforce instability and the urgency of measurable retention reform. This commentary responds to that need. Its purpose is to interrogate the policy logic underpinning the Nursing Action Initiative, highlight where current strategies remain misaligned with the structural nature of Europe's nursing workforce crisis, and propose a sharpened, retention‐driven agenda capable of delivering sustainable improvements in workforce resilience and patient care quality across the Region.

## 2 EU‐Funded “Nursing Action” Summary

The EU‐funded Nursing Action Initiative, launched by WHO/Europe in early 2025, represents the first coordinated, multi‐country effort to address the nursing shortage through an integrated policy and systems‐level approach (WHO 2025). Supported by dedicated European Union resources, the project involves 12 Member States and aims to strengthen national workforce strategies through technical assistance, structured policy dialogue, and harmonized capacity‐building efforts. Its overarching goal is to support countries in designing and implementing sustainable solutions to the persistent challenges of workforce retention, distribution, and professional development.

At its core, the Initiative is built around four interrelated priorities. First, it aims to enhance workforce intelligence by improving the accuracy, comparability, and usability of health workforce data. This area has been repeatedly identified as a bottleneck in European planning. Second, it aims to strengthen governance and strategic workforce planning, supporting Member States in aligning education, employment, and service delivery policies with current and projected population needs. Third, the Initiative commits to improving working conditions and retention mechanisms by promoting evidence‐informed policies that address workload, workplace safety, remuneration models, gender equality, and mental health protections. Fourth, it encourages innovative approaches to education and continuous professional development, particularly in relation to digital health competencies and interprofessional collaboration. This integrated approach aligns closely with the five pillars of the Framework for Action on the Health and Care Workforce in the WHO European Region 2023–2030, particularly the emphasis on retention, performance optimization, and investment (WHO 2023).

However, while the Nursing Action Initiative introduces a coherent and forward‐looking architecture for reform, its impact will depend on its ability to address long‐standing structural barriers within Member States, many of which extend beyond the health sector. This includes the persistent fragmentation of workforce governance, variability in regulatory frameworks for specialist and advanced roles, and uneven investment in working conditions across regions. Therefore, the Initiative provides a timely platform to reorient European nursing workforce policy; however, its success will ultimately require a strong political commitment, intersectoral collaboration, and measurable accountability at both national and EU levels.

## 3 The Missing Retention Architecture

The WHO has repeatedly emphasized that Europe's current workforce crisis is neither sudden nor unexpected. Instead, it reflects the accumulation of profound structural weaknesses that predated the COVID‐19 pandemic and were later amplified by it (WHO 2023). Although the Region reports “record absolute numbers” of health workers, these averages obscure critical dysfunctions in distribution, well‐being, and retention. Europe's challenge is therefore not quantitative scarcity but systemic fragility: the inability to keep nurses in the profession, protect their health, and deploy their expertise effectively.

Multiple pressures drive this instability. A significant proportion of the nursing workforce in many EU Member States is aged ≥ 55 years, pointing toward an imminent retirement cliff and the rapid erosion of clinical expertise (EUPHA‐HWR et al. 2023). The WHO has signaled that this demographic shift alone could destabilize health systems if not addressed urgently through proactive retention and succession planning (Stievano et al. 2024). Yet over decades, governance gaps, inconsistent workforce planning, and underinvestment in working conditions have created an environment in which the profession is progressively less sustainable. Retention pressures are not evenly distributed: shortages are most acute in rural, remote, and economically disadvantaged territories, where mobility toward higher‐resource systems intensifies inequity.

The implication is clear: Europe cannot train its way out of this crisis. Expanding university places remains ineffective if newly qualified nurses enter workplaces marked by unsafe staffing, limited autonomy, constrained progression pathways, and deteriorating mental and physical well‐being. Without substantial improvements in retention mechanisms, the Region will continue to lose nurses faster than they can be replaced.

In this context, the EU‐funded Nursing Action Initiative represents a welcome and potentially transformative development (WHO 2025). Its coordinated, multi‐country approach aligns with the WHO Framework for Action's strategic priorities. However, its impact will depend on whether it addresses the structural determinants of attrition that have long been overlooked. As currently configured, several critical gaps risk limiting its ability to produce durable system‐wide change.

### 3.1 Structural Gaps That Constrain Impact

The Initiative rightly recognizes that retention is central to workforce sustainability. Its focus on improving working conditions, mental health, workload management, and protection from violence is fully aligned with WHO guidance (WHO 2023, 2025). Yet this alignment is undermined by the absence of a binding retention metric. This omission leaves the central determinant of workforce sustainability ungoverned. The WHO has highlighted persistent gaps in standardized indicators of exits, vacancy rates, and intention to leave, which are core predictors of workforce collapse. Without measurable benchmarks, Member States may default to short‐term recruitment‐heavy strategies, including international recruitment from more fragile systems, thereby displacing rather than solving workforce shortages.

Education reform constitutes another major Initiative priority. The Framework for Action calls for the integration of digital health and artificial intelligence (AI) competencies, strengthened interprofessional teamwork, and robust continuous professional development pathways. These are necessary investments for a future‐ready workforce. However, increasing training capacity while mid‐career attrition accelerates is like filling a leaking bucket: it does little to counteract the immediate loss of mid‐career nurses, who are essential for mentorship, leadership, and service continuity. Educational expansion in the absence of retention safeguards risks scaling the instability rather than correcting it.

Performance optimization is equally pivotal. Redesigning multidisciplinary teams, broadening the scope of nursing practice, and promoting task‐shifting can enhance autonomy and reduce skill underutilization, which are factors consistently linked to intention to stay. Yet, although the Initiative acknowledges these needs, it lacks a concrete roadmap for operationalizing role expansion across diverse European health systems. Without governance mechanisms that enable nurses to work at the top of their license, advanced competencies remain underused, and job dissatisfaction may persist. In addition, without harmonized governance and implementation strategies, progress will remain slow and uneven.

The Initiative also addresses workforce planning, emphasizing collaboration across health, finance, and education ministries. Still, this ambition is hindered by the persistent fragmentation of data infrastructure. Many Member States lack standardized minimum datasets, reliable forecasting capacity, or mandatory reporting systems. Crucially, the EU should coordinate and support, but only national authorities can legislate and fund the conditions that determine whether nurses stay. Planning will remain aspirational without interoperable workforce intelligence that captures both public and private sectors.

While the Framework underscores workload, well‐being, and workforce safety, it does not mandate safe staffing standards. The absence of legislated nurse‐to‐patient ratios or acuity‐adjusted workload requirements leaves the strongest determinant of retention, e.g., staffing safety, outside enforceable policy in most Member States. Unsafe staffing erodes professional standards, increases adverse events, and creates moral injury, paving the way to attrition. Under such conditions, attrition will continue unchecked, regardless of improvements in recruitment or education.

Similarly, although competency strengthening and skill mix optimization are promoted, there is no unified approach to specialist and advanced nursing roles. Countries with structured advanced practice pathways demonstrate better retention and lower migration. Conversely, fragmented regulatory landscapes narrow career progression and autonomy, eliminating the very incentives that keep experienced nurses in the system and driving highly skilled nurses to seek opportunities elsewhere.

Lastly, the monitoring mechanism of the Initiative relies on voluntary participation and variable reporting. As previous WHO and EU workforce initiatives have shown (WHO 2023), voluntary accountability leads to consensus on principles but lacks durable implementation. Without enforceable standards, Europe's workforce strategy risks becoming a patchwork of uneven progress rather than a structural solution.

## 4 Policy Directions for a Retention‐Driven Workforce Sustainability Strategy

Grounded in the WHO Framework for Action and the structural deficiencies outlined above, policy efforts at both the EU and national levels must shift from a narrow emphasis on workforce expansion to a governance model in which retention becomes the primary determinant of workforce sustainability. To achieve this goal, the Nursing Action Initiative should explicitly support legislative pathways that guarantee safe staffing levels as a non‐negotiable foundation of professional well‐being and patient safety. Mandated nurse‐to‐patient ratios, ideally calibrated to patient acuity and care complexity, represent the most powerful and evidence‐based means of preventing premature exits. These standards must be complemented by robust protections against workplace violence and harassment, mandatory incident reporting, and precise mechanisms for organizational accountability, shifting the burden of safety away from individual resilience and toward structural responsibility.

Retention also requires that nurses see a future for themselves within the profession. To this end, Member States should collaborate with EU institutions to establish a coherent European framework for specialist and advanced nursing roles, harmonizing the scope of practice, regulatory authorization, and educational pathways. When career progression is fragmented or unavailable, autonomy is restricted, and highly skilled nurses are incentivized to migrate toward systems offering clearer opportunities. In contrast, well‐defined advanced roles strengthen professional identity, knowledge utilization, and intention to remain in the workforce, transforming experience into a resource rather than a liability.

Beyond career pathways, retention also depends on how effectively health systems enable nurses to apply their competencies in daily practice. Improving workforce sustainability further depends on redesigning care delivery models so that nurses can contribute at the top of their license (Stievano et al. 2024). Multiprofessional team structures, effective task‐shifting, and strengthened frontline and middle‐management leadership can reduce skill underutilization and its downstream effects on job dissatisfaction. Such reforms require not only educational investment but also governance commitments that ensure nurses are granted authority commensurate with their competencies and that decision‐making structures recognize nursing expertise as central to system performance.

To correct the cyclical instability caused by recruitment without retention—one of the structural gaps identified above—a renewed focus on strategic workforce planning is equally essential. Accurate forecasting and equitable workforce distribution can only be achieved if Member States adopt interoperable workforce intelligence systems that capture standardized indicators of exits, vacancy rates, intention to leave, and distribution across urban and rural settings. This must include data from both public and private sectors and be tied to long‐term fiscal planning. While the EU can facilitate coordination and provide technical guidance, only national authorities hold the regulatory and financial levers needed to translate intelligence into enforceable staffing and investment decisions.

Investment mechanisms must be explicitly linked to retention and equity outcomes. Funding should be directed toward interventions that demonstrably improve working conditions, stabilize staffing, and avoid the cyclical inefficiencies of recruitment without retention. Where fiscal capacity is constrained, EU‐level financial support may be required; however, eligibility should be conditional on measurable progress in staffing safety, workforce distribution, and career advancement opportunities. In this way, financial incentives become drivers of structural change rather than temporary relief.

## 5 Conclusions

The EU‐funded Nursing Action Initiative marks a significant step toward addressing Europe's long‐standing nursing workforce crisis, and its alignment with the WHO Framework provides a solid foundation for reform. Yet the core challenge remains structural. Europe's shortages are driven less by insufficient graduate numbers than by persistent failures in retention, governance, and investment. Without binding commitments to safe staffing, stronger protections for nurses, harmonized career pathways, and robust workforce data systems, the Initiative risks becoming another well‐intentioned effort without a durable system‐level impact.

To secure a sustainable nursing workforce, retention must become the central policy priority. This requires treating nursing not as a cost to be contained but as a strategic asset essential to health system resilience and societal well‐being. The Nursing Action Initiative offers a valuable opportunity. Still, its success will depend on Member States' political will to confront entrenched weaknesses and implement the structural reforms needed to retain nurses in the profession and ensure high‐quality care across the European Union. Ultimately, without a robust architecture of retention governance, neither the future of Europe's nursing workforce nor the sustainability of its health systems can be assured. A critical debate is therefore urgently needed to ensure that this Initiative evolves from aspirational coordination into enforceable, system‐level reform capable of delivering lasting improvements in European health care.

## 6 Clinical Resources

### 6.1 Health and Care Workforce in Europe: Time to Act

This 2022 report by the WHO Regional Office for Europe provides the most comprehensive assessment to date of health and care workforce challenges across the European Region, covering physicians, nurses, midwives, dentists, pharmacists, and physiotherapists (https://www.who.int/europe/publications/i/item/9789289058339).

### 6.2 Framework for Action on the Health and Care Workforce in the WHO European Region 2023–2030

The Framework, adopted in October 2023 at the 73rd Regional Committee for Europe, provides a comprehensive blueprint to address persistent and emerging workforce challenges across the 53 countries in the WHO European Region (https://www.who.int/europe/publications/i/item/EUR‐RC73‐8).

### 6.3 European Commission and WHO/Europe Sign €1.3 Million Agreement to Help EU Member States Retain and Attract More Nurses

On 6 September 2024, WHO/Europe and the European Commission signed a €1.3 million contribution agreement under the EU4Health programme (36‐month initiative) to support all 27 EU Member States in retaining current nursing staff and attracting new recruits (https://www.who.int/europe/news/item/06‐09‐2024‐european‐commission‐and‐who‐europe‐sign‐‐1.3‐million‐agreement‐to‐help‐eu‐member‐states‐retain‐and‐attract‐more‐nurses).

### 6.4 WHO/Europe Launches EU‐Funded “Nursing Action” Project to Address Nursing Shortages in the EU

On 17 January 2025, WHO/Europe, in partnership with the European Commission, officially launched “Nursing Action,” the first EU‐funded, multi‐country initiative explicitly aimed at tackling critical shortages in the nursing workforce across the European Union (https://www.who.int/europe/news‐room/events/item/2025/01/17/default‐calendar/who‐europe‐launches‐eu‐funded‐‐nursing‐action‐‐project‐to‐address‐nursing‐shortages‐in‐the‐eu).

## Conflicts of Interest

The authors declare no conflicts of interest.

## Data Availability

Data sharing not applicable to this article as no datasets were generated or analysed during the current study.
